# Towards Information-Theoretic Security and Privacy in IoT: A Three-Factor AKA Protocol Supporting Forgotten Password Reset

**DOI:** 10.3390/e28020205

**Published:** 2026-02-11

**Authors:** Yicheng Yu, Kai Wei, Hongtu Li, Kai Zhang

**Affiliations:** 1 School of Electronic and Communication Engineering, Shenzhen Polytechnic University, Shenzhen 518055, China; 2 College of Computer Science and Technology, Jilin University, Chaoyang District, Changchun 130012, China; 3 Department of Computer Science and Engineering, National Chung Hsing University, Taichung 402, Taiwan

**Keywords:** Internet of Things, authentication, security protocol, forgotten password reset

## Abstract

The growth of the Internet of Things (IoT) has created many problems. A wise example is presented by the design of secure, efficient authentication and key agreement (AKA) protocols. A novel three-factor AKA protocol for the IoT is presented in this paper. The scheme integrates password, biometric, and device-based factors that achieved strong security, which gives anonymity to the user, achieves forward secrecy, and makes the scheme resilient to various attacks like replay, impersonation, and de-synchronization. It also adds a safe lost-password-reset functionality, which makes the protocol more usable. Security analysis proves its strength against the typical adversary, while performance evaluation shows that the solution is better than existing solutions in terms of computational and communication efficiency. The work proposes a practical and scalable security solution for IoT systems, which satisfies the high security standard but within the constraints of an IoT system.

## 1. Introduction

The Internet of Things (IoT) is part of the new generation of information technology. This thing–thing interconnection system forms an intelligent programme. The IoT combines massive sensor and intelligent terminal communication technology [1]. The essence of the world of things—a comprehensive perception of the environment, reliable transmission of mass data, and intelligent processing of information—makes it possible to control and manage objects. The IoT is a key engine driving economic and societal digital transformation and high-quality development, as the application scenarios extend from smart homes to industrial manufacturing, smart cities, environmental monitoring, intelligent healthcare, and other critical fields.

However, these conveniences and increased efficiencies also bring with them serious security challenges due to the rapid proliferation and deep use of the IoT [2]. In this situation, reliable security systems are no longer optional, but necessary for the system to be trusted. Secure access control mechanisms that rely on reliable identity authentication play a critical role in preventing unauthorized data theft and malicious command injection.

Usually, IoT systems exhibit heavy resource asymmetry; there are powerful cloud servers and resourceful user terminals alongside a huge number of extremely resource-scarce sensor nodes. Due to limited computing power and battery life, the latter finds it difficult to bear the overhead of traditional cryptographic protocols [3]. Because of this reality, lightweight security schemes must be designed for heterogeneous environments [2]. The three-layer model (User–Gateway–Sensor Node) is an almost universally adopted model that uses the gateway as a trusted third party to offload the authentication burden. However, many existing schemes that are based on this model still suffer from serious drawbacks. Some are efficient but sacrifice security. Thus, they fail to achieve the basic properties like user anonymity, forward secrecy, and so on. Two properties should be achieved, and at the same time, they suffer from high latency. Other schemes, while achieving these security properties, suffer from other attacks such as gateway bypass, node capture, etc. [4].

In this work, we address the above issues and propose a new authentication and key agreement protocol that is intended to be used in IoT three-layer architecture for usefulness–security trade-off. The proposed protocol integrates lightweight security provisions that enable it to efficiently run on devices with limited resources without compromising the security strength required for sensitive applications, unlike existing schemes. It also provides a password reset and recovery system that is useful when users forget their passwords.

The principal contributions of this work can be summarized as follows:

- We present a mutual authentication and key agreement scheme that facilitates the establishment of a secure session by the user with the sensor node through the gateway, significantly reducing their overhead on sensor nodes.
- We can show by both formal and informal security analysis that the suggested protocol can withstand replay, impersonation, de-synchronization attacks, and user anonymity; forward secrecy is guaranteed in this scheme.
- In comparison with state-of-the-art schemes, the protocol has overall advantages regarding security attributes and performance metrics. Due to its low communication and computational overhead, it is suitable for large-scale resource-constrained IoT environments.
- A secure password-reset procedure is designed to handle user scenarios in case they forget their passwords. Moreover, the usability and robustness of the protocol have been enhanced for actual deployments.

The remainder of this paper is organized as follows: Section 2 reviews related work on IoT authentication protocols. Section 3 introduces the system model, threat assumptions, and cryptographic foundations. Section 4 elaborates on the detailed steps of the proposed protocol. Security analysis and performance evaluation are conducted in Section 5 and Section 6, respectively. Finally, Section 7 concludes the paper and outlines future research directions.

## 2. Related Work

From a methodological perspective, existing authentication protocols for WSN systems can be broadly categorized into two-factor authentication and three-factor AKA designs, depending on their assumed security scope and guaranteed properties. In this work, we focus on three-factor AKA protocols that operate under comparable system models and security objectives, including (i) explicit session key agreement for subsequent secure communications, (ii) design for a user–gateway–sensor architecture, where the gateway supports resource-constrained sensor nodes, and (iii) practical usability features such as secure credential update or recovery mechanisms. For instance, Yu et al. proposed a relevant prior two-factor authentication protocol with formal verification [5]. While such schemes constitute important early contributions, they do not target a comparable security scope to three-factor AKA protocols—e.g., they do not provide explicit session key agreement, PUF-based device binding, forgotten-password recovery, or resilience against desynchronization attacks. Therefore, as these two-factor schemes are not designed for the three-factor AKA setting (password, smart card, and biometrics), we exclude them from the set of baseline schemes in our security and performance evaluations to maintain fairness, consistency of underlying assumptions, and comparability of results. The baseline inclusion criteria are summarized in Appendix A.

In recent years, research on Authentication and Key Agreement (AKA) protocols for the Internet of Things (IoT) and Wireless Sensor Networks (WSN) has predominantly focused on lightweight three-factor designs, hardware-based root-of-trust mechanisms (e.g., Physical Unclonable Functions, PUFs), and formal security verification under strong adversarial models. Central to these efforts is the use of lightweight cryptographic techniques that facilitate the tightly coupled derivation of session keys from password, device, and biometric factors. Additionally, the incorporation of dynamic identities and the use of one-time random numbers have become key strategies for achieving a balance between high security and low computational overhead in resource-constrained devices. The security of these protocols is typically proven using formal methods, such as BAN logic, AVISPA, and ProVerif, which help mitigate security risks and ensure the robustness of the underlying protocol design [6,7,8,9].

The effort of Sahoo et al. in three-factor authentication with ECC [6] provides a bidirectional authentication scheme with dynamic identities, thus achieving low communication overhead and optimized sensor load. The proposed scheme of Sahoo et al. is useful for 5G WSNs and IoT systems that need to support high concurrency and low latency. Security proofs are according to the Random oracle model. Huang [7] suggested an ECC-based three-factor AKA scheme for general WSNs, which was shown to be secure against impersonation, and session-specific, stolen-verifier, and replay attacks as proven through BAN logic and ProVerif. Moreover, it was shown to be resistant to other attacks. Vinoth et al. [8] introduced a multifactor authentication scheme in the industrial IoT space, which allows using multiple devices to access the IoT environment. After a detailed analysis performed by Sun [10], it was found that it was vulnerable to impersonation, replay, and desynchronization attacks. Following this, the authors modified their existing protocol. Likewise, multi-gateway WSNs and hierarchical gateway architectures have also inspired the proposed techniques for improved cross-domain roaming, high availability, and efficient communication. These methods feature optimizations to balance the number of communication rounds and the load on edge nodes [11]. Moreover, various works tried to reduce the computational cost of ECC by using non-traditional number-theoretic foundations. One three-factor protocol based on Chebyshev chaotic mappings has been proposed, with security proofs in the RoR and BAN models emphasizing the freshness of the session [12].

To achieve noise robustness and privacy protection, biometrics are embedded into authentication protocols. The use of fuzzy extractors and auxiliary data is at the heart of this application, which transforms unstable biometric templates into stable key shares that contribute to session key generation. This mechanism effectively reduces the chance of linkability and template leakage, which are important security concerns in three-factor authentication schemes. When it comes to privacy-sensitive applications like healthcare, they include other mechanisms like honey lists and controlled disclosure to strike a balance between anonymity and accountability, enabling features like local password updates and resilient recovery from device loss. The use of hardware-based trust models is also on the rise. This is especially true when it comes to PUFs integrated into AKA protocols. PUFs offer a key material and a physically unclonable identity that is generated from the random microvariations that transpired during device manufacturing. This is very useful against device cloning, physical capture, and side-channel attacks. It also functions well in a hostile environment. In the IoMT and in medical WSNs, PUFs have been embedded into a three-factor AKA protocol, where CRPs bind session keys with its unique hardware feature. Thus, they are able to greatly reduce the risk of static keys being stored and device replication. These solutions also focus on tackling key optimization problems such as CRP management, environmental robustness, and error correction to make PUF-based solutions applicable in ultra-low-power devices [13,14,15,16].

Continued systematic cryptanalysis and iterative enhancement of existing protocols are critical directions for improving the security of AKA protocols. The analysis by Kumar et al. [17] on various three-factor schemes showed that substantial loss of smart card security is possible with offline password guessing attacks, insider attacks, and de-synchronization attacks. As per their findings, they proposed a modified version of the protocol, which had security proofs and performance evaluations in the random oracle model. The following studies have strengthened these protocols with a focus on user anonymity, unlinkability, device-side state, key rotation, and other important aspects. These improvements give complete security against adversaries who can make use of session key leaks and temporary secrets [10,18].

## 3. Preliminaries

### 3.1. System Model

The Internet of Things authentication system model has three main participants, which are the user, the gateway node, and the sensor node. By rationally partitioning functions, this model resolves conflicts between the resource constraints and security requirements for IoT [19].

The user is the ultimate requestor of data, initiating data access requests to sensor nodes with devices such as mobile terminals. Sensor nodes undertake the collection of environmental data and are usually constrained by resources; in particular, limited computation capability, memory capacity, and energy availability. The gateway node is the system’s safe central core of the system. Having processing power far superior to sensor nodes, the gateway node takes care of the registration of both users and sensor nodes. In addition, it helps the user and sensor node achieve mutual authentication and secure communication.

**Figure 1:**
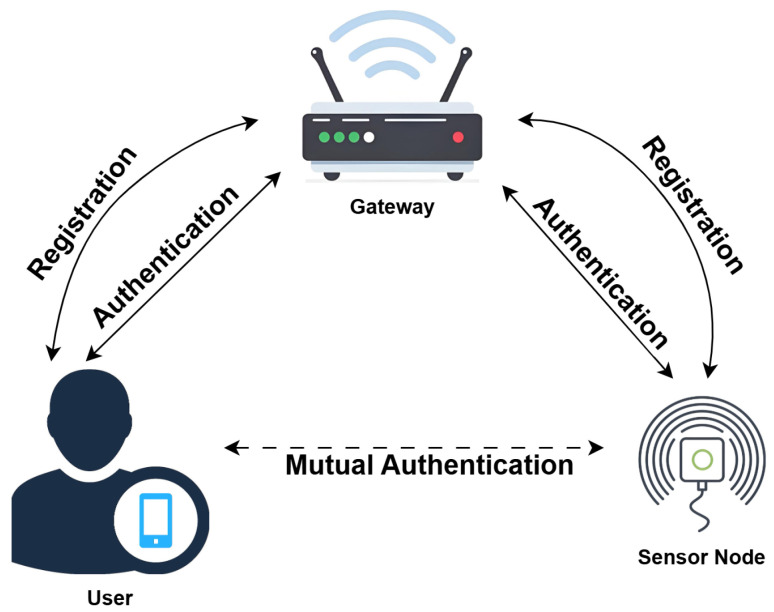
System model.

Under the model, the user needs to authenticate with the sensor node before starting their communication. The authentication process encompasses an offline registration phase, wherein the user and the sensor node provide their registration details to the gateway node separately. This gateway generates and securely delivers initial authentication credentials for the devices. When a user initiates an access request, they first submit their authentication information to the gateway. Once verified by the gateway, it helps mutually authenticate the user and the target sensor node. The successful authentication not only verifies the legitimacy of the communicating parties, but also enables the user and sensor node to negotiate a temporary session key. This session key keeps any following data transmissions private and unaltered. The authentication process of this model is shown in Figure 1.

The primary advantage of this model lies in the rationality of its architectural design. The gateway node, acting as a trusted intermediary, does the heavy lifting of the authentication process, while the very resource-constrained sensor nodes are only tasked with simple computations. This division of labor satisfies the low power consumption requirements of IoT terminals while also enabling reliable identity authentication and key agreement, thus providing a practical and feasible security foundation for large-scale IoT applications [20].

### 3.2. Adversary Model

To precisely evaluate the security of the authentication protocol, it is essential to explicitly define the adversary’s capabilities, thereby establishing a formal adversary model. Although the Dolev-Yao model [21] serves as a standard for such analysis, stricter assumptions regarding the adversary’s power are necessary to address emerging threats like side-channel attacks [22]. Accordingly, we define an adversary with the following capabilities:

- The adversary can perform offline exhaustive attacks on the user identity space and the weak password space.
- The adversary can extract sensitive information stored in entities such as smart cards via means like side-channel analysis.
- The adversary has full control over the public channel, meaning they can eavesdrop on, intercept, tamper with, replay, or block any message in transit.
- When evaluating an n-factor authentication protocol, it is assumed that the adversary has compromised n-1 authentication factors. This assumption is used solely to assess the protocol’s robustness under partial credential leakage, and it does not imply that the smart cards, passwords, or biometrics are always considered unprotected during normal operation.
- The adversary may have obtained a temporary session key from a past session through other means. For evaluating forward secrecy, it is assumed that the adversary has obtained the long-term private key(s) of one or more parties.

### 3.3. Fuzzy Extractor (FE)

Intra-class noise refers to the natural variations in the biometric signature of the same user. Fuzzy extractors are meant to remove this noise, such that a biometric input that is nearly the same can produce the exact same output. Its operational principle consists of two core phases:

- The Generation function $GEN\left(Bio_{i}\right)=\left(b_{i},par_{i}\right)$: This function takes the user’s initial biometric template $Bio_{i}$ as input. It generates a secret output $b_{i}$ and a public auxiliary data $par_{i}$.
- The Reproduction function $REP\left(Bio_{i}^{\prime },par_{i}\right)=b_{i},dis\left(Bio_{i}^{\prime },Bio_{i}\right)\leq ▵t$: This function takes a new biometric sample $Bio_{i}^{\prime }$, and public auxiliary data $par_{i}$ as input during the subsequent verification. As long as $Bio_{i}^{\prime }$ is close enough to the original $Bio_{i}$, the function can reproduce the same secret output $b_{i}$ which means that the biometric reading can tolerate minor variations.

### 3.4. Physical Unclonable Function (PUF)

A Physical Unclonable Function, often referred to as a PUF, is a security mechanism that employs distinct and unavoidable microscopic physical variations that occur during semiconductor fabrication, giving every physical entity a unique digital fingerprint. Usually integrated in integrated circuits, PUF is a physically observable unconventional response generated by the intrinsic physical characteristics when stimulated by an input challenge signal, denoted as Re=PUF(Ch). The response to this challenge is determined by the internal physical structure of the chip. It gives the chip a very high degree of uniqueness and randomness. This results in chips made in the same batch having a unique PUF response. The fact that these devices do not rely on any specific secure storage makes them ideal for lightweight action, as performed in the case of IoT sensor nodes for the purposes of device authentication and key derivation.

## 4. Proposed Scheme

The proposed protocol primarily comprises the following phases: initialization, user registration, sensor node registration, and authentication login. In addition, auxiliary phases for forgotten password reset and password/biometric update are designed. For ease of reading, the main symbols used in the proposed protocol are summarized in Appendix B.

### 4.1. Initialization Phase

The gateway GWN establishes two tables in its secure memory: TableSensornodeInfo for storing sensor node parameters and TableUserInfo for user credentials. GWN then selects a system master key τ, specifies a symmetric encryption scheme $E_{k}\left(\cdot \right)/D\left(\cdot \right)$, and designates cryptographic hash functions h(·) and H(·), where H(·) is reserved for fuzzy verification on the user side.

### 4.2. User Registration Phase

**Figure 2:**
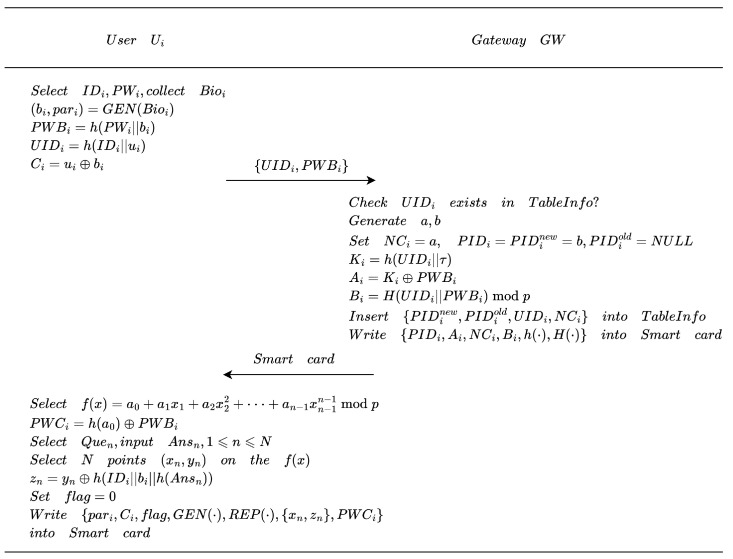
User registration phase.

Figure 2 illustrates the user registration process, with the specific steps as follows:

1. The user $U_{i}$ selects the identity $ID_{i}$ and the password $PW_{i}$, and collects biometric information $Bio_{i}$. $U_{i}$ selects a random number $u_{i}$, and computes $\left(b_{i},par_{i}\right)=GEN\left(Bio_{i}\right)$, $PWB_{i}=h(PW_{i}\left|\right|b_{i})$, $UID_{i}=h(ID_{i}\left|\right|u_{i})$, and $C_{i}=u_{i}⊕b_{i}$, then transmits the registration information $\left\{UID_{i},PWB_{i}\right\}$ to the gateway node GWN via a secure channel. The secure channel during the registration phase can be realized through practical means, such as an out-of-band channel, pre-shared credentials during device provisioning, or physical access at deployment time.
2. Upon receiving the user registration information, GWN verifies whether $UID_{i}$ exists in TableUserInfo. If a match is found, GWN rejects the registration request. Otherwise, GWN selects two random numbers *a* and *b*, assigns $NC_{i}=a$ and $PID_{i}=PID_{i}^{new}=b$, sets $PID_{i}^{old}=NULL$, and computes $K_{i}=h(UID_{i}||\tau )$, $A_{i}=K_{i}⊕PWB_{i}$, and $B_{i}=H(UID_{i}||PWB_{i})\;\mathrm{mod}\;p$. GWN then inserts the tuple $\left\{PID_{i}^{new},PID_{i}^{old},UID_{i},NC_{i}\right\}$ into TableUserInfo. Finally, GWN writes $\left\{PID_{i},A_{i},NC_{i},B_{i},h\left(\cdot \right),H\left(\cdot \right)\right\}$ to a smart card SC and sends it to $U_{i}$ through a secure channel.
3. Upon receiving the registration response, $U_{i}$ randomly selects a polynomial $f\left(x\right)=a_{0}+a_{1}x_{1}+a_{2}x_{2}^{2}+\cdots +a_{N-1}x_{N-1}^{N-1}\;\mathrm{mod}\;p$ of degree N−1 and computes $PWC_{i}=h\left(a_{0}\right)⊕PWB_{i}$. Subsequently, $U_{i}$ chooses *N* security questions $\left\{Que_{n},1⩽n⩽N\right\}$ with corresponding answers $\left\{Ans_{n},1⩽n⩽N\right\}$, selects *N* distinct points $\left\{\left(x_{n},y_{n}\right),1⩽n⩽N\right\}$ on the polynomial f(x), and computes secret shares $z_{n}=y_{n}⊕h(ID_{i}\left|\right|b_{i}||h\left(Ans_{n}\right))$ for each point. Finally, $U_{i}$ initializes a state flag as flag=0 and stores $\left\{par_{i},C_{i},flag,GEN\left(\cdot \right),REP\left(\cdot \right),\left\{\left(x_{n},z_{n}\right),1⩽n⩽N\right\},PWC_{i}\right\}$ in SC.

Notably, the number of security questions *N* should be selected by balancing security strength and usability in practical IoT deployments. A small value of *N* reduces user burden but may weaken resistance against guessing or social engineering attacks, while an excessively large *N* increases cognitive load and degrades user experience, especially for long-lived or frequently accessed IoT systems. In typical scenarios, we recommend choosing *N* in the range of 4 to 6, which provides sufficient entropy for secure password recovery while maintaining acceptable usability.

### 4.3. Sensor Node Registration Phase

**Figure 3:**
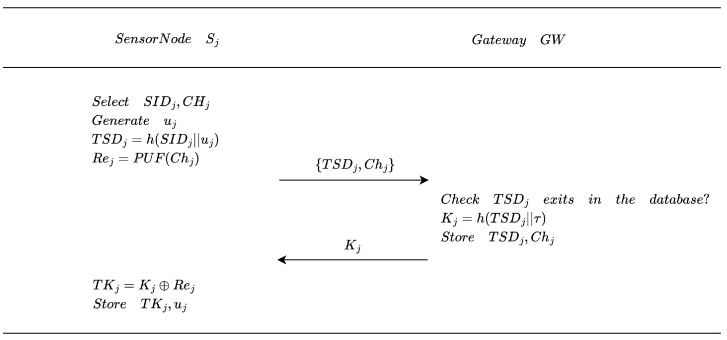
Sensor node registration phase.

The sensor node $S_{j}$ selects the identity $SID_{j}$ and a challenge $Ch_{j}$, generates a random number $u_{j}$, and computes $TSD_{j}=h(SID_{j}\left|\right|u_{j})$. Using the PUF function, it generates the response $Re_{j}=PUF\left(Ch_{j}\right)$. Subsequently, $S_{j}$ transmits $TSD_{j}$ and $Ch_{j}$ to the gateway GW. GW checks whether $TSD_{j}$ duplicates any record in the database. If no duplicates are found, it computes $K_{j}=h(TSD_{j}||\tau )$, stores $TSD_{j}$ and $Ch_{j}$, and transmits $K_{j}$ back to $S_{j}$. Upon receiving $K_{j}$, $S_{j}$ calculates $TK_{j}=K_{j}⊕Re_{j}$ and stores $TK_{j}$ and $u_{j}$. The process of sensor node registration is shown in Figure 3.

### 4.4. Authentication Phase

During the authentication phase, messages are exchanged among the user, the gateway node, and the sensor node. To prevent replay attacks, upon receiving a message, the receiving entity is required to follow the Common Message Acceptance Rule (CMAR). For any received authentication message carrying a timestamp $T_{k}$, the receiver performs described as follows:

1. Freshness: check $|T_{now}-T_{k}|\leq ▵T$; otherwise reject.
2. Anti-replay (strict ordering): maintain a lightweight last-accepted timestamp record $T_{last}$ for the sender and reject if $T_{k}\leq T_{last}$.
3. Authentication: verify the message-specific authenticator.
4. Upon successful verification, update $T_{last}=T_{k}$; otherwise reject. The record $T_{last}$ is updated only after successful authentication and is initialized to 0.

**Figure 4:**
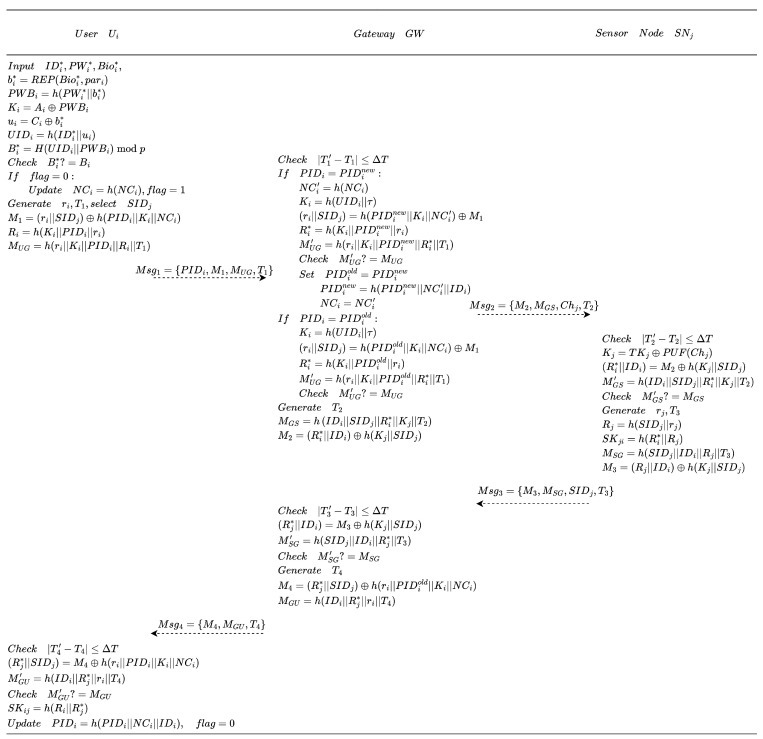
Authentication phase (all receivers additionally apply the common message acceptance rule for freshness and replay prevention).

The process during the authentication phase is shown in Figure 4, and the specific steps are as follows:

1. $U_{i}$ inserts SC into a terminal and enters $ID_{i}$, $PW_{i}$, and provides biometric input $Bio_{i}$. SC computes values $b_{i}^{*}=REP\left(Bio_{i}^{*},par_{i}\right)$, ${PWB}_{i}=h(PW_{i}^{*}\left|\right|b_{i}^{*})$, $K_{i}=A_{i}⊕PWB_{i}$, $u_{i}=C_{i}⊕b_{i}^{*}$, $UID_{i}=h(ID_{i}^{*}\left|\right|u_{i})$, and $B_{i}^{*}=H(UID_{i}||PWB_{i})\;\mathrm{mod}\;p$, then verifies whether $B_{i}^{*}$ equals the stored value $B_{i}$. If the verification fails, SC terminates the session. Otherwise, it checks the state flag. If flag=0, SC computes $NC_{i}=h\left(NC_{i}\right)$ and updates flag=1. Subsequently, SC selects the identity $SID_{j}$ of the target sensor node to be accessed, generates a random number $r_{i}$, acquires the current timestamp $T_{1}$, and computes values $M_{1}=(r_{i}||SID_{j})⊕h(PID_{i}||K_{i}||NC_{i})$, $R_{i}=h(K_{i}||PID_{i}\left|\right|r_{i})$, and $M_{UG}=h(r_{i}\left|\right|K_{i}||PID_{i}\left|\right|R_{i}\left|\right|T_{1})$. Finally, SC sends the message $Msg_{1}=\left\{PID_{i},M_{1},M_{UG},T_{1}\right\}$ to GWN.
2. Upon receiving the user login request, GWN processes it according to the common message acceptance rule (CMAR) described above. Then, GWN searches the TableUserInfo for a $\left(PID_{i}^{new},PID_{i}^{old}\right)$ pair and operates according to the following rules:
  - If a pair exists where $PID_{i}=PID_{i}^{new}$, GWN retrieves the corresponding $UID_{i}$ and $NC_{i}$, computes values $NC_{i}^{\prime }=h\left(NC_{i}\right)$, $K_{i}=h(UID_{i}||\tau )$, $\left(r_{i}||SID_{j})=h(PID_{i}^{new}\left|\right|K_{i}||NC_{i}^{\prime }\right)⊕M_{1}$, $R_{i}^{*}=h(K_{i}||PID_{i}^{new}\left|\right|r_{i})$, and $M_{UG}^{\prime }=h(r_{i}\left|\right|K_{i}||PID_{i}^{new}\left|\right|R_{i}^{*}\left|\right|T_{1})$, and verifies whether $M_{UG}^{\prime }$ equals $M_{UG}$. If the check fails, GWN terminates the session and rejects the login request. Otherwise, GWN performs the reassignments $PID_{i}^{old}=PID_{i}^{new}$, $PID_{i}^{new}=h(PID_{i}^{new}||NC_{i}^{\prime }||ID_{i})$, and $NC_{i}=NC_{i}^{\prime }$.
  - If a pair exists where $PID_{i}=PID_{i}^{old}$, GWN retrieves the corresponding $UID_{i}$ and $NC_{i}$, computes values $K_{i}=h(UID_{i}||\tau )$, $\left(r_{i}||SID_{j})=h(PID_{i}^{old}\left|\right|K_{i}||NC_{i}\right)⊕M_{1}$, $R_{i}^{*}=h(K_{i}||PID_{i}^{old}\left|\right|r_{i})$, and $M_{UG}^{\prime }=h(r_{i}\left|\right|K_{i}||PID_{i}^{old}\left|\right|R_{i}^{*}\left|\right|T_{1})$, and verifies whether $M_{UG}^{\prime }$ equals $M_{UG}$. If the check fails, GWN terminates the session and rejects the login request.
  - If no pair exists where either $PID_{i}=PID_{i}^{new}$ or $PID_{i}=PID_{i}^{old}$, GWN terminates the session and rejects the login request.
  After completing the aforementioned operations, GWN acquires a new timestamp $T_{2}$ and queries TableSensorNodeInfo using $SID_{j}$ to retrieve the corresponding $K_{j}$ and $Ch_{j}$. GWN then computes $M_{GS}=h(ID_{i}||SID_{j}\left|\right|R_{i}^{*}\left|\right|K_{j}\left|\right|T_{2})$ and $M_{2}=(R_{i}^{*}||ID_{i})⊕h(K_{j}||SID_{j})$, and subsequently transmits the message $Msg_{2}=\left\{M_{2},M_{GS},Ch_{j},T_{2}\right\}$ to the sensor node $S_{j}$.
3. Upon receiving the message from GWN, $S_{j}$ first processes it according to the common message acceptance rule (CMAR) described above. Subsequently, $S_{j}$ computes values $K_{j}=TK_{j}⊕PUF\left(Ch_{j}\right)$, $\left(R_{i}^{*}||ID_{i}\right)=M_{2}⊕h(K_{j}||SID_{j})$, and $M_{GS}^{\prime }=h(ID_{i}||SID_{j}\left|\right|R_{i}^{*}\left|\right|K_{j}\left|\right|T_{2})$, and subsequently verifies whether $M_{GS}^{\prime }$ equals $M_{GS}$. If the verification fails, the session is terminated. If successful, $S_{j}$ generates a new random number $r_{j}$, acquires a fresh timestamp $T_{3}$, and computes values $R_{j}=h(SID_{j}\left|\right|r_{j})$, $SK_{ji}=h(R_{i}^{*}\left|\right|R_{j})$, $M_{SG}=h(SID_{j}||ID_{i}\left|\right|R_{j}\left|\right|T_{3})$, and $M_{3}=(R_{j}||ID_{i})⊕h(K_{j}||SID_{j})$. Finally, $S_{j}$ transmits the response message $Msg_{3}=\left\{M_{3},M_{SG},SID_{j},T_{3}\right\}$ back to GWN.
4. Upon receipt of the message from $S_{j}$, GWN processes it according to the common message acceptance rule (CMAR) described above. GWN then computes $\left(R_{j}^{*}||ID_{i}\right)=M_{3}⊕h(K_{j}||SID_{j})$ and $M_{SG}^{\prime }=h(SID_{j}||ID_{i}\left|\right|R_{j}^{*}\left|\right|T_{3})$, and checks whether the locally computed $M_{SG}^{\prime }$ equals the received $M_{SG}$. If not, GWN terminates the session. Otherwise, GWN acquires a new timestamp $T_{4}$ and computes $M_{4}=(R_{j}^{*}||SID_{j})⊕h(r_{i}||PID_{i}^{old}\left|\right|K_{i}||NC_{i})$ and $M_{GU}=h(ID_{i}\left|\right|R_{j}^{*}\left|\right|r_{i}\left|\right|T_{4})$. Finally, GWN transmits the response message $Msg_{4}=\left\{M_{4},M_{GU},T_{4}\right\}$ back to $U_{i}$.
5. Following the receipt of the authentication response, $U_{i}$ processes it according to the common message acceptance rule (CMAR) described above. Subsequently, $U_{i}$ computes $(R_{j}^{*}||SID_{j})=M_{4}⊕h(r_{i}||PID_{i}\left|\right|K_{i}||NC_{i})$ and $M_{GU}^{\prime }=h(ID_{i}\left|\right|R_{j}^{*}\left|\right|r_{i}\left|\right|T_{4})$. If the equivalence $M_{GU}^{\prime }=M_{GU}$ is confirmed, the mutual authentication is deemed successful and a session key $SK_{ij}=h(R_{i}\left|\right|R_{j}^{*})$ is established. To conclude the process, $U_{i}$ assigns $PID_{i}=h(PID_{i}||NC_{i}||ID_{i})$ and resets flag=0.

### 4.5. Forgotten Password Reset Phase

If user $U_{i}$ forgets his password $PW_{i}$, $U_{i}$ initiates the reset process by entering the identity $ID_{i}$, providing the biometric $Bio_{i}$, and inserting the smart card SC. $U_{i}$ then sequentially submits the answers $\left\{Ans_{n},1⩽n⩽N\right\}$ to the *N* security questions. Following this, $U_{i}$ computes $b_{i}=REP\left(Bio_{i},par_{i}\right)$ and $y_{n}=z_{n}⊕h(ID_{i}\left|\right|b_{i}||h(Ans_{n}))$. Upon correct response to all *N* security questions, $U_{i}$ successfully reconstructs the polynomial $f\left(x\right)=a_{0}+a_{1}x_{1}+a_{2}x_{2}^{2}+\cdot \cdot \cdot +a_{n-1}x_{n-1}^{n-1}\;\mathrm{mod}\;p$, computes $f\left(0\right)=a_{0}$, and executes operations $PWB_{i}=h\left(a_{0}\right)⊕PWC_{i}$ and $u_{i}=C_{i}⊕b_{i}$. Subsequently, $U_{i}$ enters a new password $PW_{i}^{new}$, computes $PWB_{i}^{new}=h(PW_{i}^{new}\left|\right|b_{i})$, $A_{i}^{new}=A_{i}⊕PWB_{i}⊕PWB_{i}^{new}$, $B_{i}^{new}=H(h(ID_{i}\left|\right|u_{i})||PWB_{i}^{new})\;\mathrm{mod}\;p$, and $PWC_{i}^{new}=h\left(a_{0}\right)⊕PWB_{i}^{new}$, and finally updates SC by overwriting the existing $A_{i}$, $B_{i}$, and $PWC_{i}$ with new values $A_{i}^{new}$, $B_{i}^{new}$, and $PWC_{i}^{new}$.

### 4.6. Password/Biometrics Update Phase

$U_{i}$ inserts SC into the terminal and enters $ID_{i}$ and $PW_{i}$, and provides biometric $Bio_{i}$. SC executes a verification algorithm to authenticate $U_{i}$’s identity, consistent with the process described in the login and authentication phase. If the verification is successful, $U_{i}$ then inputs a new password $PW_{i}^{new}$, provides a new biometric $Bio_{i}^{new}$, and computes values $\left(b_{i}^{new},par_{i}^{new}\right)=GEN\left(Bio_{i}^{new}\right)$, $PWB_{i}^{new}=h(PW_{i}^{new}\left|\right|b_{i}^{new})$, $C_{i}^{new}=C_{i}⊕b_{i}⊕b_{i}^{new}$, $A_{i}^{new}=A_{i}⊕PWB_{i}⊕PWB_{i}^{new}$, and $B_{i}^{new}=H(UID_{i}||PWB_{i}^{new})\;\mathrm{mod}\;p$. Subsequently, $U_{i}$ randomly selects a new private polynomial, $f^{\prime }\left(x\right)=a_{0}^{\prime }+a_{1}^{\prime }x_{1}+a_{2}^{\prime }x_{2}^{2}+\cdots +a_{N-1}^{\prime }x_{N-1}^{N-1}\;\mathrm{mod}\;p$ of degree N−1, chooses *N* new security questions $\left\{Que_{n}^{new},1⩽n⩽N\right\}$ with corresponding answers $\left\{Ans_{n}^{new},1⩽n⩽N\right\}$, selects *N* distinct points $\left\{\left(x_{n}^{new},y_{n}^{new}\right),1⩽n⩽N\right\}$ on the polynomial $f^{\prime }\left(x\right)$, and computes secret shares $z_{n}^{new}=y_{n}^{new}⊕h(ID_{i}\left|\right|b_{i}^{new}||h\left(Ans_{n}^{new}\right))$ for each point. Furthermore, $U_{i}$ updates the SC by overwriting the existing stored data with these newly computed values.

## 5. Security Analysis

### 5.1. Correctness Verification

To formally verify the correctness of the authentication protocol, we employ BAN logic [23], which was introduced by Burrows, Abadi, and Needham in 1989. BAN logic is widely adopted in the analysis of authentication and key agreement protocols for IoT and wireless sensor networks due to its clear abstraction of authentication beliefs, message freshness, and key establishment goals. Its interpretability makes it particularly suitable for reasoning about mutual authentication and session key agreement in resource-constrained IoT environments. This logic characterizes authentication goals using modal operators, and its associated formal notation and rules provide the foundational methodology for protocol verification, as specified in Table 1. These definitions are generic and not specific to the proposed scheme.

The verification process commences with the idealization of the protocol messages and the postulation of initial assumptions. Following this, the authentication goals are formalized. The process concludes with the application of logical inference rules to satisfy all defined goals. The corresponding details for the idealization, assumptions/goals, and derivations are provided in Table 2, Table 3, and Table 4, respectively.

### 5.2. Informal Security Analysis

We demonstrate that the proposed protocol has critical security features and demonstrates resilience against a range of well-known attacks.

#### 5.2.1. Anonymity and Untraceability

During the login and authentication phase, an adversary may intercept messages transmitted over public channels among the user, the gateway node, and sensor nodes. However, in the proposed protocol, none of the messages generated throughout this phase contain the user’s identity identifier $U_{i}d$. Consequently, the attacker cannot obtain any identity-related information, thus ensuring the anonymity of the user. Furthermore, the pseudo-identity is updated after each session, and random numbers $r_{i}$ and $r_{j}$ are regenerated randomly per session. Thus, for the same user, the information transmitted over the public channel differs between sessions, making it infeasible for an adversary to determine whether two distinct sessions originate from the same user. As a result, the proposed protocol also achieves untraceability of the user.

#### 5.2.2. Mutual Authentication

The formal verification results using BAN logic indicate that the user and the sensor node are convinced of the authenticity of each other and the session key. This result provably verifies that the proposed protocol successfully achieves mutual authentication.

#### 5.2.3. Session Key Agreement

According to the protocol description, the user and the sensor node collaboratively establish a session key $SK_{ij}=h(R_{i}\left|\right|R_{j})$ during the authentication phase, which will be used for their subsequent secure communication.

#### 5.2.4. Perfect Forward Secrecy

Assume that an attacker accidentally captures $U_{i}$’s long-term private key $K_{i}$, the nonce $NC_{i}$, $S_{j}$’s long-term private key $K_{j}$, and GWN’s long-term private key τ, in addition to intercepting previously transmitted information over the public channel. In the proposed protocol, the session key is calculated as $SK_{ij}=h(R_{i}\left|\right|R_{j}),R_{i}=h(K_{i}||PID_{i}\left|\right|r_{i}),(r_{i}||SID_{j})=h_{1}\left(PID_{i}\left|\right|K_{i}||NC_{i}\right)⊕M_{1}$, where the session key is determined by the nonce $NC_{i}$ of the current session. However, $NC_{i}$ is updated via a hash function after each session. As a result, even if the attacker obtains the current $NC_{i}$, the one-way nature of the hash function prevents them from deriving the nonce $NC_{i}$ from previous sessions, thus making it impossible to compute past session keys. Therefore, the proposed protocol ensures forward security effectively.

#### 5.2.5. N-Factor Security

In this subsection, we analyze a worst-case scenario in which the adversary compromises N−1 authentication factors, including the smart card via side-channel attacks, to evaluate the robustness of the proposed three-factor design. Among the three security factors considered in the protocol, the password is cryptographically weaker than the smart card and the biometric. Consider a scenario in which an adversary obtains the smart card (extracting its data through a side-channel attack) and also steals the user’s biometric. The adversary could then attempt to guess the identity $ID_{i}^{*}$ and password $PW_{i}^{*}$, and calculate $b_{i}=REP\left(Bio_{i},par_{i}\right)$, $PWB_{i}^{*}=h(PW_{i}^{*}\left|\right|b_{i})$, $K_{i}^{*}=A_{i}⊕PWB_{i}^{*}$, $u_{i}=C_{i}⊕b_{i}$, $UID_{i}^{*}=h(ID_{i}^{*}\left|\right|u_{i})$ and $B_{i}^{*}\overset{?}{=}H(UID_{i}^{*}||PWB_{i}^{*})\;\mathrm{mod}\;p$. Due to the fuzzy verification mechanism, there are approximately 10,000 candidate pairs $\left(ID_{i}^{*},PW_{i}^{*}\right)$ that can satisfy the verification condition. Distinguishing the correct credentials from this set would require the adversary to perform about 10,000 online login attempts, which is computationally prohibitive. Moreover, such a high volume of attempts can be easily detected and blocked by GWN.

#### 5.2.6. Forgotten Password Reset

The proposed protocol incorporates a secure forgotten password recovery function. A user who has forgotten their password can regain access only by correctly providing their identities, biometrics, and correct answers to all security questions. Successful verification of these factors allows the reconstruction of the polynomial f(x) and grants the authorization to reset the password.

#### 5.2.7. Resistance Against Man-in-the-Middle (MITM) Attack

The analysis of the Man-in-the-Middle (MITM) attack relies on the results derived from BAN logic (as discussed in Section 4), which formalizes the mutual authentication process in the proposed protocol. This analysis demonstrates that the protocol can effectively prevent MITM attacks by ensuring that both the user and sensor node authenticate each other through a secure session establishment process.

#### 5.2.8. Resistance Against Replay Attack

Each authentication message ($Msg_{1}$–$Msg_{4}$) carries a timestamp and includes a hash-based authenticator ($M_{UG}$, $M_{GS}$, $M_{SG}$ and $M_{GU}$), so any modification will be detected. In addition to the freshness check $|T_{now}-T_{k}|\leq ▵T$, the protocol adopts the common message acceptance rule (CMAR): each receiver maintains a lightweight last-accepted timestamp record for its peer and rejects any message with a non-increasing timestamp (i.e., $T_{k}\leq T_{last}$). The last-accepted record is updated only after successful authentication. Therefore, any replay of $Msg_{1}$–$Msg_{4}$ will be detected and rejected, even if replayed within ▵T. Consequently, replay attacks are effectively prevented.

#### 5.2.9. Resist Known Session-Specific Temporary Information Attack

$U_{i}$ and $S_{j}$ successfully negotiate and establish a session key $SK_{ij}=h(R_{i}\left|\right|R_{j})=h(h(K_{i}||PID_{i}\left|\right|r_{i})||h(SID_{j}\left|\right|r_{j}))$. Even if an adversary obtains the temporary random numbers $r_{i}$ and $r_{j}$ from the current session, the correct session key $SK_{ij}$ cannot be calculated without knowing the long-term secret $K_{i}$ of $U_{i}$. Therefore, the proposed protocol is resistant to known session-specific temporary information attacks.

#### 5.2.10. Resistance Against De-Synchronization Attack

To effectively resist de-synchronization attacks, it is crucial to maintain synchronization between $U_{i}$ and GWN, particularly for the pseudo-identity $PID_{i}$ and $NC_{i}$. The proposed protocol incorporates a flag on the $U_{i}$ side and stores the last two pseudo-identifiers ($PID_{i}^{new}$ and $PID_{i}^{old}$) for each user on the GWN side to preserve synchronization. The mechanism for maintaining synchronization is explained in two attack scenarios. First, if A blocks the authentication message $Msg_{1}$, $U_{i}$ will update $NC_{i}$ and set flag=1, while GWN, not having received $Msg_{1}$, will not update its $NC_{i}$, leading to a temporary state of de-synchronization. However, since flag=1, $U_{i}$ will not increment $NC_{i}$ again in the subsequent login request. Upon receiving this request, GWN can resynchronize by computing $NC_{i}=h\left(NC_{i}\right)$, thus restoring the consistency of the state. Second, if A intercepts the message $Msg_{4}$, GWN completes the update of $PID_{i}^{new}$ (with $PID_{i}^{old}$ recording the previous value), but $U_{i}$, not having received $Msg_{4}$, cannot update $PID_{i}$. When $U_{i}$ initiates a new session, GWN will detect that the submitted $PID_{i}$ matches the stored $PID_{i}^{old}$. This identifies a de-synchronization attempt, triggering a resynchronization of $PID_{i}$ and $NC_{i}$ according to Step 2 of the authentication phase, which recovers protocol state consistency. In summary, through the flag mechanism and the dual pseudo-identity storage strategy, the protocol can maintain or recover synchronization between the user and the gateway even after message interception, effectively resisting de-synchronization attacks.

To ensure a fair and meaningful evaluation, we compare our protocol with four representative and recent three-factor AKA schemes that share similar system models and security objectives, while schemes operating under different authentication factors or security scopes are excluded from the comparative analysis. Table 5 compares the security properties achieved by our proposed protocol with those of four recently counterparts.

## 6. Performance Analysis

Given that the registration phase for both the user and the sensor node is a one-time operation, and password/biometric updates are infrequent, the performance comparison focuses on the authentication phases.

### 6.1. Computational Performance Analysis

The computational performance comparison is based on counting the cryptographic operations executed by each entity during one complete authentication session. The following notations are used: $T_{h}$, $T_{f}$, $T_{puf}$, $T_{ed}$ and $T_{M}$, representing the time cost of a single hash operation, fuzzy extractor operation, PUF operation, symmetric encryption/decryption, and elliptic curve point multiplication, respectively. The time consumption for string concatenation and XOR operations is considered insignificant and is ignored. We evaluated the computational overhead following the experimental environment described by Wu et al. [13], which utilized a platform with the following specifications: an Intel(R) Core(TM) i7-13700K CPU (5.40 GHz), 32.0 GB RAM, with Windows 10 OS. The execution times for various operations are summarized in Table 6, indicating that the fuzzy extractor function requires a running time similar to that of an ECC point multiplication operation [24].

During the authentication phase of the scheme proposed by Wu et al. [13], the user performs six hash operations and one fuzzy extraction operation when initiating a login request. After receiving the response from the gateway node GWN, the user further performs three hash operations. Therefore, the total computational cost at the user side consists of nine hash operations and one fuzzy extraction operation. Upon receiving the user’s login request, the GWN executes eight hash operations, two PUF operations, and one fuzzy extraction operation. After obtaining the response message from the sensor node $S_{j}$, the GWN additionally performs four hash operations. As a result, the total computational overhead at the GWN amounts to twelve hash operations, two PUF operations, and one fuzzy extraction operation. Meanwhile, after receiving message $M_{2}$, the sensor node performs a total of six hash operations, one PUF operation, and one fuzzy extraction operation.

In the authentication phase of the scheme introduced by Sahoo et al. [6], the user incurs four hash operations, one fuzzy extraction operation, one elliptic curve point multiplication, and one symmetric encryption when generating the login request. Upon receiving the reply from the gateway node GWN, the user carries out two additional hash operations and one symmetric decryption. Hence, the overall computational burden at the user side includes six hash operations, one fuzzy extraction operation, one elliptic curve point multiplication, and two symmetric cryptographic operations. At the gateway node, four hash operations, one symmetric encryption, one symmetric decryption, and one elliptic curve point multiplication are executed upon processing the user’s login request. Subsequently, after the response from the sensor node $S_{j}$ is received, the GWN performs two further hash operations along with one elliptic curve point multiplication. Accordingly, the cumulative computational cost at the GWN amounts to six hash operations, two symmetric cryptographic operations, and two elliptic curve point multiplications. For the sensor node, once the message from the gateway node is obtained, the authentication procedure requires seven hash operations, two elliptic curve point multiplications, one symmetric encryption, and one symmetric decryption in total.

With respect to the authentication phase in the scheme presented by Huang et al. [7], the user executes seven hash operations, one fuzzy extraction operation, and three elliptic curve point multiplications while constructing the login request. After the feedback from the gateway node GWN is received, the user additionally conducts ten hash operations and one elliptic curve point multiplication. Consequently, the user-side computation involves a total of seventeen hash operations, one fuzzy extraction operation, and four elliptic curve point multiplications. On the gateway side, processing the user’s login request requires ten hash operations together with one elliptic curve point multiplication. Once the response from the sensor node $S_{j}$ is obtained, the GWN proceeds to carry out seven further hash operations and an additional elliptic curve point multiplication. Therefore, the overall computational effort at the GWN amounts to seventeen hash operations and two elliptic curve point multiplications. From the perspective of the sensor node, upon reception of the message from the gateway node, the authentication procedure entails eight hash operations and three elliptic curve point multiplications in total.

For the authentication procedure in the scheme developed by Kumar et al. [17], the user carries out five hash operations, one fuzzy extraction operation, and two symmetric encryption operations when submitting the login request. After receiving the return message from the gateway node GWN, the user performs two additional hash operations and two symmetric decryption operations. Accordingly, the total computational requirement at the user side consists of seven hash operations, one fuzzy extraction operation, and four symmetric cryptographic operations. At the gateway node, two symmetric encryption operations and one symmetric decryption operation are executed during the processing of the user’s login request. Following the reception of the response from the sensor node $S_{j}$, the GWN conducts two hash operations together with one symmetric encryption and one symmetric decryption. Hence, the aggregate computational cost incurred by the GWN includes five hash operations and five symmetric cryptographic operations. As for the sensor node, once the message forwarded by the gateway node is received, a total of four hash operations, one symmetric encryption, and one symmetric decryption are required to complete the authentication process.

In the authentication phase of our proposed scheme, the user computes seven hash operations and one fuzzy extraction operation while initiating the login request. Upon receiving the response from the gateway node GWN, the user further computes four hash operations. As such, the overall computational load at the user side is limited to eleven hash operations and one fuzzy extraction operation. For the gateway node, eight hash operations are required to process the user’s login request, followed by four additional hash operations after the response from the sensor node $S_{j}$ is obtained. Consequently, the total computational cost incurred by the GWN amounts to twelve hash operations. Regarding the sensor node, after receiving the message from the gateway node, the authentication procedure involves six hash operations and one PUF operation in total.

This comparative analysis of computational overhead, presented in Table 7, shows that our proposed protocol outperforms four recent counterparts [6,7,13,17].

### 6.2. Communication Performance Analysis

In this subsection, we compare the communication performance of the proposed protocol with the four protocols mentioned above. The communication performance comparison considers only the authentication messages exchanged over the public channel. The bit lengths of all parameters and message components are explicitly defined, and the total communication overhead is obtained by summing the sizes of all transmitted messages. The bit widths for the parameters in the protocol are defined as follows: random number (256 bits), hash value (256 bits), PUF challenge (128 bits), identity (128 bits), elliptic curve point (256 bits), and timestamp (32 bits). Concurrently, the output of the symmetric encryption is defined as an integer multiple of 128 bits (i.e., its block size).

In the authentication phase of the scheme proposed by Wu et al. [13], a total of four messages, namely $M_{1}=\left\{HID_{i},R_{1},V_{1},T_{1}\right\}$, $M_{2}=\left\{SID_{j},R_{2},TPW_{i},Ch_{j},V_{2},T_{2}\right\}$, $M_{3}=\left\{R_{3},V_{3},T_{3}\right\}$, and $M_{4}=\left\{R_{4},V_{4},T_{4}\right\}$, are transmitted among the participating entities. In these messages, $HID_{i}$, $V_{1}$, $V_{2}$, $V_{3}$ and $V_{4}$ denote hash values, while $T_{1}$, $T_{2}$, $T_{3}$ and $T_{4}$ represent timestamps. In addition, $SID_{j}$ corresponds to the identity of the sensor node, and $Ch_{j}$ denotes the PUF challenge. The values $R_{1}$, $R_{2}$, $TPW_{i}$, $R_{3}$ and $R_{4}$ are obtained through XOR operations, whose lengths are determined by the longer operands involved, resulting in bit-lengths of 256 bits, 512 bits, 256 bits, 256 bits and 512 bits, respectively. Consequently, the total size of the transmitted information in this scheme amounts to 265∗5+32∗4+128+128+256+512+256+256+512=3456 bits.

In the authentication phase of the scheme proposed by Sahoo et al. [6], four messages, denoted as $\left\{M_{2},M_{4},UID_{i},T_{1}\right\}$, $\left\{G_{1},G_{2},G_{5},T_{2}\right\}$, $\left\{S_{2},S_{4},S_{5},S_{6},T_{3}\right\}$, and $\left\{S_{5},S_{6},T_{4}\right\}$, are exchanged during the authentication procedure. In these messages, $M_{2}$, $UID_{i}$, $G_{2}$, $S_{4}$ and $S_{6}$ represent hash values, whereas $T_{1}$, $T_{2}$, $T_{3}$ and $T_{4}$ correspond to timestamps. Moreover, $S_{2}$ denotes a point on the elliptic curve. The value $G_{1}$ is generated through a XOR operation, whose length is determined by the longer operand and is equal to 256 bits. In addition, $M_{4}$, $G_{5}$ and $S_{5}$ are the outputs of symmetric encryption. According to the lengths of their corresponding plaintexts, the sizes of $M_{4}$, $G_{5}$ and $S_{5}$ are 512 bits, 1024 bits and 128 bits, respectively. Consequently, the total amount of transmitted data in this scheme is 265∗6+32∗4+256+256+512+1024+128∗2=3968 bits.

In the authentication phase of the scheme proposed by Huang et al. [7], four messages, namely $\left\{R_{u},D_{2},D_{3},TID_{i},T1\right\}$, $\left\{R_{u},R_{g},D_{4},D_{5},D_{6},D_{7},T_{2}\right\}$, $\left\{R_{s},D_{9},D_{10},T_{3}\right\}$, and $\left\{R_{s},e_{i},D_{10},D_{11},D_{12},D_{13},D_{14},T_{4}\right\}$, are transmitted during the authentication process. In these messages, $R_{u}$, $R_{g}$ and $R_{s}$ denote points on the elliptic curve, while $D_{3}$, $TID_{i}$, $D_{7}$, $D_{9}$, $D_{10}$ and $D_{14}$ represent hash values. In addition, $T_{1}$, $T_{2}$, $T_{3}$ and $T_{4}$ are timestamps. The values $D_{2}$, $D_{4}$, $D_{5}$, $D_{6}$, $e_{i}$, $D_{11}$, $D_{12}$ and $D_{13}$ are generated through XOR operations, whose bit-lengths are determined by the longer operands involved and are equal to 512 bits, 256 bits, 256 bits, 256 bits, 256 bits, 256 bits, 256 bits and 256 bits, respectively. Consequently, the total amount of transmitted information in this scheme is 256∗5+256∗7+32∗4+512+256+256+256+256+256+256+256=5504 bits.

In the authentication phase of the scheme proposed by Kumar et al. [17], four messages, denoted as $\left\{B_{4},B_{5},B_{6},TS_{1}\right\}$, $\left\{B_{7},B_{9},TS_{2}\right\}$, $\left\{B_{10},B_{11},TS_{3}\right\}$ and $\left\{B_{11},B_{12},TS_{4}\right\}$, are exchanged during the authentication process. In these messages, $TS_{1}$, $TS_{2}$, $TS_{3}$ and $TS_{4}$ represent timestamps, while $B_{7}$ and $B_{10}$ denote hash values. In addition, $B_{4}$, $B_{5}$, $B_{9}$, $B_{11}$ and $B_{12}$ are the outputs of symmetric encryption, whose lengths are integer multiples of 128 bits. Based on the lengths of the corresponding plaintexts, the sizes of $B_{4}$, $B_{5}$, $B_{9}$, $B_{11}$ and $B_{12}$ are 640 bits, 1152 bits, 1408 bits, 896 bits and 896 bits, respectively. The value $B_{6}$ is generated through a XOR operation, and its bit-length is determined by the longer operand involved, resulting in a length of 1152 bits. Consequently, the total amount of transmitted information in this scheme is 32∗4+256∗2+640+1152+1408+896∗2+896+1152=7680 bits.

During the authentication phase, the proposed protocol exchanges a total of four messages ($Msg_{1}$ to $Msg_{4}$). The composition of $Msg_{1}$ is as follows:

- $PID_{i}$: User’s pseudo-identity, 128 bits.
- $M_{1}$: Generated by XORing the concatenation of the random number $r_{i}$ and the sensor node’s identity $SID_{j}$ with a hash value (using cyclic padding). Its length equals the sum of the bit lengths of $r_{i}$ and $SID_{j}$, i.e., 256 bits + 128 bits.
- $M_{UG}$: Hash output, 256 bits.
- $T_{1}$: Timestamp, 32 bits.

The length of $Msg_{1}$ is 800 bits. The lengths of $Msg_{2}$, $Msg_{3}$, and $Msg_{4}$ are subsequently derived as 800, 800, and 672 bits, respectively. Consequently, the aggregate communication cost for the authentication phase is 3072 bits. A comparison with four recent relevant protocols, presented in Table 8, further confirms that our protocol holds an advantage in communication overhead.

## 7. Discussion

The proposed protocol for three-factor authentication and key agreement seeks to resolve security and efficiency issues in the Internet of Things (IoT) environment. The protocol enables secure communication in extremely constrained settings, which are often seen in the IoT, by using lightweight cryptographic operations. The protocol enjoys a high level of security, as the integration of Physical Unclonable Functions (PUFs) for device authentication and fuzzy extractors for biometric authentication have low cost. This is essential for applications that require both safety and real-time performance in industrial IoT and healthcare systems.

This protocol also features a secure and easy-to-use password reset procedure, which is a useful solution to the problem of lost passwords in IoT systems. The reset was structured to combine biometric verification with security questions and will ensure that accounts can be reset without exposing anyone to the system. The importance of this feature significantly increases in case the user has to interact with such a device for a long time in large-scale IoT deployments.

A follow-up stage of research could include assessments of how well the protocol can operate in environments with more capable computing resources. Furthermore, integrating additional trust models, such as decentralized identities or blockchain-based authentication, could further enhance the protocol’s security and scalability in more complex IoT ecosystems.

## 8. Conclusions

This work proposes a new three-factor authentication and key agreement protocol for the IoT that solves security and performance problems in the resource-constrained environment. The protocol employs password, biometric and device-based authentication for enhanced security features. Furthermore, user anonymity, forward secrecy, and other attacks are resistant to it. Additionally, enabling a secure forgotten password reset feature enhances the overall user experience while maintaining a high level of security. The proposed scheme is more efficient than the existing schemes and has been shown by performance evaluations to be practical for large-scale IoT deployment.

The analysis and the comparison of performance of the protocol show that it is useful to provide reliable and scalable security services for IoT systems. Nonetheless, additional optimization of other trust models for high-capacity IoT devices can also be referenced from future works. In summary, this work provides a secure, efficient, and scalable solution that will aid in advancing the state of security in the IoT.

## Figures and Tables

**Table 1:** Standard BAN logic notations and inference rules.

**Symbol**	**Description**
P∣≡X	*P* believes *X*
P◃X	*P* sees *X*
P∣∼X	*P* once said *X*
#(X)	*X* is fresh
P∣⇒X	*P* has jurisdiction over *X*
(X,Y)	The combination of *X* and *Y*
${\left(X\right)}_{K}$	Encrypt *X* in some form using *K* as the key
$P\overset{SK}{↔}Q$	*P* and *Q* share the key *K*
**Formula Symbol**	**Rule**
$\frac{P|\equiv \left(P\overset{K}{↔}Q\right),P◃{\left(X\right)}_{K}}{P|\equiv Q|∼X}$	Message meaning rule (MMR)
$\frac{P|\equiv \#(X)}{P|\equiv \#(X,Y)}$	Freshness conjunction rule (FCR)
$\frac{P|\equiv \#(X),P|\equiv Q|∼X}{P|\equiv Q|\equiv X}$	Nonce verification rule (NVR)
$\frac{P|\equiv Q|\Rightarrow X,P|\equiv Q|\equiv X}{P|\equiv X}$	Jurisdiction rule (JR)
$\frac{P|\equiv Q|\equiv (X,Y)}{P|\equiv Q|\equiv X}\;\;\;\frac{P|\equiv X,P|\equiv Y}{P|\equiv (X,Y)}$	Belief conjunction rule (BCR)

**Table 2:** Idealized forms.

Message	Idealized Forms
$M_{1}$	$U_{i}\to GWN:{\left(r_{i},SID_{j}\right)}_{U_{i}\overset{\left(K_{i},NC_{i}\right)}{⟷}GWN}$
$M_{2}$	$GWN\to S_{j}:{\left(R_{i},ID_{i}\right)}_{GWN\overset{K_{j}}{⟷}S_{j}}$
$M_{3}$	$S_{j}\to GWN:{\left(R_{j},ID_{i}\right)}_{S_{j}\overset{K_{j}}{⟷}GWN}$
$M_{4}$	$GWN\to U_{i}:{\left(R_{j}\right)}_{GWN\overset{\left(PID_{i},K_{i},NC_{i}\right)}{⟷}U_{i}}$

**Table 3:** Assumptions and goals.

**No.**	**Assumptions**
$A_{1}$	$U_{i}|\equiv \#\left(r_{i},r_{j}\right)$
$A_{2}$	$S_{j}|\equiv \#\left(r_{i},r_{j}\right)$
$A_{3}$	$GWN|\equiv \#(r_{i},r_{j})$
$A_{4}$	$U_{i}|\equiv U_{i}\overset{\left(PID_{i},ID_{i},K_{i},NC_{i}\right)}{⟷}GWN$
$A_{5}$	$GWN|\equiv U_{i}\overset{\left(PID_{i},ID_{i},K_{i},NC_{i}\right)}{⟷}GWN$
$A_{6}$	$GWN|\equiv GWN\overset{K_{j}}{⟷}S_{j}$
$A_{7}$	$GWN|\equiv S_{j}\overset{K_{j}}{⟷}S_{j}$
$A_{8}$	$U_{i}|\equiv S_{j}\Rightarrow \left(U_{i}\overset{SK}{⟷}S_{j}\right)$
$A_{9}$	$S_{j}|\equiv U_{i}\Rightarrow \left(U_{i}\overset{SK}{⟷}S_{j}\right)$
**No.**	**Goals**
$G_{1}$	$U_{i}|\equiv \left(U_{i}\overset{SK}{↔}S_{j}\right)$
$G_{2}$	$U_{i}|\equiv S_{j}|\equiv \left(U_{i}\overset{SK}{↔}S_{j}\right)$
$G_{3}$	$S_{j}|\equiv \left(U_{i}\overset{SK}{↔}S_{j}\right)$
$G_{4}$	$S_{j}|\equiv U_{i}|\equiv \left(U_{i}\overset{SK}{↔}S_{j}\right)$

**Table 4:** Analysis based on BAN logic.

No.	Statement	Premise and Rule
$s_{1}$	$GWN◃{\left(r_{i},SID_{j}\right)}_{U_{i}\overset{\left(K_{i},NC_{i}\right)}{⟷}GWN}$	$M_{1}$
$s_{2}$	$GWN|\equiv U_{i}|∼{\left(r_{i},SID_{j}\right)}_{U_{i}\overset{\left(K_{i},NC_{i}\right)}{⟷}GWN}$	$s_{1},A_{5},MMR$
$s_{3}$	$GWN|\equiv U_{i}|\equiv {\left(r_{i},SID_{j}\right)}_{U_{i}\overset{\left(K_{i},NC_{i}\right)}{⟷}GWN}$	$s_{2},A_{3},NVR$
$s_{4}$	$S_{j}◃{\left(r_{i},ID_{i}\right)}_{GWN\overset{K_{j}}{⟷}S_{j}}$	$M_{2}$
$s_{5}$	$S_{j}|\equiv GWN|∼{\left(R_{i},ID_{i}\right)}_{U_{i}\overset{K_{j}}{⟷}GWN}$	$s_{4},A_{7},MMR$
$s_{6}$	$S_{j}|\equiv \#\left(R_{i}\right)$	$A_{2},FCR,R_{i}=h(K_{i}||PID_{i}\left|\right|r_{i})$
$s_{7}$	$S_{j}|\equiv GWN|\equiv {\left(R_{i},ID_{i}\right)}_{U_{i}\overset{K_{j}}{⟷}GWN}$	$s_{5},s_{6},NVR$
$s_{8}$	$S_{j}|\equiv U_{i}|\equiv \left(U_{i}\overset{SK}{↔}S_{j}\right)\;\begin{array}{c} G_{4} \end{array}$	$s_{3},s_{7},SK_{ij}=h(R_{i}\left|\right|R_{j})$
$s_{9}$	$S_{j}|\equiv \left(U_{i}\overset{SK}{↔}S_{j}\right)\;\begin{array}{c} G_{3} \end{array}$	$s_{8},A_{9},JR$
$s_{10}$	$GWN◃{\left(R_{j},ID_{i}\right)}_{S_{j}\overset{K_{j}}{⟷}GWN}$	$M_{3}$
$s_{11}$	$GWN|\equiv S_{j}|∼{\left(R_{j},ID_{i}\right)}_{S_{j}\overset{K_{j}}{⟷}GWN}$	$s_{10},A_{6},MMR$
$s_{12}$	$GWN|\equiv S_{j}|\equiv {\left(R_{j},ID_{i}\right)}_{S_{j}\overset{K_{j}}{⟷}GWN}$	$s_{11},A_{3},NVR,R_{j}=h(SID_{j}\left|\right|r_{j})$
$s_{13}$	$U_{i}◃{\left(R_{j}\right)}_{GWN\overset{\left(PID_{i},K_{i},NC_{i}\right)}{⟷}U_{i}}$	$M_{4}$
$s_{14}$	$U_{i}|\equiv GWN|∼{\left(R_{j}\right)}_{GWN\overset{\left(PID_{i},K_{i},NC_{i}\right)}{⟷}U_{i}}$	$s_{13},A_{4},MMR$
$s_{15}$	$U_{i}|\equiv GWN|\equiv {\left(R_{j}\right)}_{GWN\overset{\left(PID_{i},K_{i},NC_{i}\right)}{⟷}U_{i}}$	$s_{14},A_{1},NVR,R_{j}=h(SID_{j}\left|\right|r_{j})$
$s_{16}$	$U_{i}|\equiv S_{j}|\equiv \left(U_{i}\overset{SK}{↔}S_{j}\right)\;\begin{array}{c} G_{2} \end{array}$	$s_{12},s_{15},SK_{ij}=h(R_{i}\left|\right|R_{j})$
$s_{17}$	$U_{i}|\equiv \left(U_{i}\overset{SK}{↔}S_{j}\right)\;\begin{array}{c} G_{1} \end{array}$	$s_{16},A_{8},JR$

**Table 5:** Comparison of security features.

	Wu et al. [13]	Sahoo et al. [6]	Huang et al. [7]	Kumar et al. [17]	Ours
Anonymity	✔	✔	✔	✔	✔
Untraceability	×	×	✔	✔	✔
Mutual authentication	✔	✔	✔	✔	✔
Session key agreement	✔	✔	✔	✔	✔
Perfect forward secrecy	×	✔	✔	×	✔
N-factor security	×	×	✔	✔	✔
Forgotten password reset	×	×	×	×	✔
Resistance against MITM attack	✔	✔	✔	✔	✔
Resistance against replay attack	✔	✔	✔	✔	✔
Resist known session-specific temporary information attack	✔	✔	✔	×	✔
Resistance against de-synchronization attack	✔	✔	✔	✔	✔

**Table 6:** Computation times for operations (ms).

Symbol	Computation Time (ms)
$T_{h}$	0.0083
$T_{f}$	6.0926
$T_{M}$	6.0926
$T_{ed}$	5.6396
$T_{puf}$	1.9741

**Table 7:** Computational overhead comparison (ms).

Protocol	User	Sensor Node	Gateway
Wu et al. [13]	$9T_{h}+T_{f}\approx 6.17$	$6T_{h}+T_{f}+T_{puf}\approx 8.12$	$12T_{h}+2T_{f}+T_{puf}\approx 14.26$
Sahoo et al. [6]	$6T_{h}+T_{f}+2T_{ed}+T_{M}\approx 23.51$	$7T_{h}+2T_{ed}+2T_{M}\approx 23.52$	$6T_{h}+2T_{ed}+2T_{M}\approx 23.51$
Huang et al. [7]	$17T_{h}+T_{f}+4T_{M}\approx 30.60$	$8T_{h}+3T_{M}\approx 18.34$	$17T_{h}+2T_{M}\approx 12.33$
Kumar et al. [17]	$7T_{h}+4T_{ed}+T_{f}\approx 28.71$	$4T_{h}+2T_{ed}\approx 11.31$	$5T_{h}+5T_{ed}\approx 28.24$
Ours	$11T_{h}+T_{f}\approx 6.18$	$6T_{h}+T_{puf}\approx 2.02$	$12T_{h}\approx 0.10$

**Table 8:** Communication overhead comparison.

Protocol	Rounds	Communication Overhead
Wu et al. [13]	4	3456 bits
Sahoo et al. [6]	4	3968 bits
Huang et al. [7]	4	5504 bits
Kumar et al. [17]	4	7680 bits
Ours	4	3072 bits

## Data Availability

Data is contained within the article.

## Appendix A. Baseline Inclusion Criteria for Comparative Evaluation

To avoid unfair comparisons across fundamentally different security objectives and system assumptions, a protocol is included as a baseline in Table 5, Table 7 and Table 8 only if it satisfies all of the following criteria:

- It is explicitly formulated as an AKA protocol with a defined session key establishment objective for subsequent secure communications.
- It adopts a three-factor authentication setting (password, biometric and smart card), or an explicitly equivalent three-factor model.
- It is designed for the User–Gateway–Sensor architecture (or an explicitly comparable three-party WSN setting), where the gateway assists resource-constrained sensor nodes.
- It considers practical usability mechanisms relevant to long-lived deployments, such as secure credential update or recovery.

These criteria are introduced to ensure that the comparative evaluation focuses on protocols that share comparable goals and assumptions with our three-factor AKA design.

## Appendix B. Protocol Notations

**Table A1:** Notations used in the proposed protocol.

Symbol	Description	Symbol	Description
$U_{i}$	User *i*	$S_{j}$	Sensor node *j*
GWN	Gateway node	$ID_{i}$	Identity of user $U_{i}$
$SID_{j}$	Identity of sensor node $S_{j}$	$PID_{i}$	Pseudo-identity of user $U_{i}$
$PW_{i}$	Password of user $U_{i}$	$Bio_{i}$	Biometric information of user $U_{i}$
$b_{i}$	Biometric key derived from the fuzzy extractor	$par_{i}$	Public helper data for the fuzzy extractor
$K_{i}$	Long-term secret key of user $U_{i}$	$K_{j}$	Long-term secret key of sensor node $S_{j}$
$NC_{i}$	Nonce counter associated with user $U_{i}$	$r_{i},r_{j}$	Session random numbers generated by $U_{i}$ and $S_{j}$
$SK_{ij}$	Session key shared between $U_{i}$ and $S_{j}$	$Ch_{j}$	Challenge input to the PUF
$Re_{j}$	Response generated by the PUF	h(·),H(·)	Cryptographic hash functions
GEN/REP	Fuzzy extractor generation and reproduction functions	*N*	Number of security questions for password recovery
